# Phenotypic Effects and Inhibition of Botrydial Biosynthesis Induced by Different Plant-Based Elicitors in *Botrytis cinerea*

**DOI:** 10.1007/s00284-017-1399-3

**Published:** 2017-11-17

**Authors:** Eva Liñeiro, Antonio J. Macias-Sánchez, Marisa Espinazo, Jesús M. Cantoral, Javier Moraga, Isidro G. Collado, Francisco J. Fernández-Acero

**Affiliations:** 1 Microbiology Laboratory, Andalusian Center for Grape and Grapevine Research, Faculty of Marine and Environmental Sciences, University of Cadiz, Polígono Rio San Pedro s/n, 11510 Puerto Real, Cádiz, Spain; 2 Organic Chemistry Department, Faculty of Science, University of Cadiz, Puerto Real, Cádiz, Spain

**Keywords:** *Botrytis cinerea*, Plant-based elicitor, Carbon sources, *Botrytis* patterning phenotype, Phytotoxin inhibition

## Abstract

*Botrytis cinerea* is considered a model organism for the study of plant–pathogen interaction showing great genetic diversity and a high degree of morphological variability depending on environmental conditions. The use of new compounds and plant-based elicitors may trigger the expression of different *B. cinerea* genes, providing new sources of virulence factors. This work is focused on elucidating the phenotypic effect in *B. cinerea* of different carbon sources such as glucose, cellulose and tomato cell walls (TCW). Production of botrydial and dihydrobotrydial toxins was evaluated using thin-layer chromatography (TLC), proton nuclear magnetic resonance spectroscopy (^1^H-NMR) and mass spectrometry (UPLC-HRESIMS). Expression of the toxin biosynthesis gene *BcBOT2* was followed using RT-qPCR. Results show an inhibition of the toxin biosynthesis pathway when TCW are present as a sole carbon source, suggesting that the toxin is only produced when rich molecules, like glucose, are available for fungal metabolism. That suggests a connection between gene expression of virulence factors and environmental conditions, where the silent genes can be induced by different culture conditions.

**Electronic supplementary material:**

The online version of this article (10.1007/s00284-017-1399-3) contains supplementary material, which is available to authorized users.

## Introduction

Phytopathogenic fungi *Botrytis cinerea* is the causal agent of grey mould, which produces fungal disease in more than 200 commercially important crops around the world. It is also significant because of the role it plays as a model in the plant–pathogen system: it has been placed in second position in the list of “top ten” fungal plant pathogens [7]. The characterization of its genome sequence in 2011 [1] made possible the development of several different molecular approaches. Most of these molecular approaches have been directed to elucidating the molecular mechanisms underlying plant–pathogen interactions and so identifying those genes/proteins involved in the virulence [4]. In the “post-genomic era”, new high-throughput methodologies have provided the capacity to collect an enormous amount of biological information. The latest genomic assembly of *B. cinerea*, generated by Wageninggen University and Sygenta in February 2015, has provided us with 11,700 predicted protein-coding genes and 12,140 coding transcripts [16], and recent proteomic approaches make available a new set of potential virulence factors. Nevertheless, taken together, in all the published proteomics data on *Botrytis cinerea* obtained to date, the number of known *Botrytis* proteins covered only a very small proportion (less than 10%) of the predicted proteins in the annotated fungal genome. The improvement of this functional annotation is crucial for a more comprehensive insight into the biology of the fungi. *B. cinerea* strains are highly versatile and adaptable to different host and environmental conditions. This versatility is objectified in different pathogenicity/virulence factors, used by the fungus according to the particular situation. Thus, the experimental growth conditions are crucial for the results obtained. It is well known in the proteomics research community that the output depends on the input [2]. This approach is characteristic of several recent proteomics studies, for example, on cellulose [12], pectin-degrading enzymes [20] and on the early secretome [11] in *B. cinerea*. Most of these studies have been performed by introducing modifications to the assay and culture conditions, including the presence of different carbon sources and plant extracts. These substances, known as plant-based elicitor, act as elicitors that induce a virulent stage in the fungus and produce a dramatic change in the fungal response [13]. Thus, the use of new compounds and plant-based elicitors may trigger the expression of different and/or new sets of *B. cinerea* genes, providing new sources of potential virulence factors.

Plant-based elicitors also are considered to be a promising strategy for environmentally friendly crop protection. In plants, elicitors are capable of stimulating plant defense responses and elicit specific defenses that help the plant to develop resistance against pathogen attacks [8]. This response has been classified as the microbe-associated molecular patterns (MAMPs) of chemical elicitors, whereby plant resistance is induced by microbe-derived molecules; pathogen-associated molecular patterns (PAMPs) are induced specifically by molecules from pathogenic microbes. On the other hand, as a result of pathogen invasion, plant-derived substances produced damage-associated molecular patterns (DAMPs) [23]. DAMP-inducing substances are mainly associated with the degradation of plant cell walls; oligogalacturonides (OGs) that trigger a MAP kinase cascade in *A. thaliana* are an example [23]. However, in spite of the well-known effects that plant elicitors produce to enhance plant protection, their effects in pathogen biology remain to be elucidated.

The main aim of the present research is to clarify the changes in the *B. cinerea* phenotype induced by different carbon sources and plant-based elicitors that have previously been proven to trigger a fungal response, including glucose, cellulose and deproteinized tomato fruits [13]. First, we collected information from classical microbiology approaches and measured the influence that those elicitors produce on colonies, hyphal morphology, conidial production and growth rates, during dark and light periods. Second, we study the effect of these compounds on secondary metabolism at the level of toxin production. Specifically, in botrydial biosynthesis a metabolite associated with the phytopathogenic process [6]. Finally, a molecular biology study was conducted to unravel the differential gene expression obtained with each compound. Our results show crucial changes in the phenotypes observed, suggesting a connection between the gene expression of the virulence factors and culture conditions.

## Materials and Methods

### Microorganisms and Culture Conditions

*Botrytis cinerea* strain 2100 was obtained from the Spanish Type Culture Collection (http://www.cect.org). Conidial stock suspension was prepared by culturing the fungus on potato dextrose agar (PDA) plates for 12 days at 22 °C. Spores were collected by gentle mixing with NaCl 0.9% (w/v) on the surface of mature conidiophores and filtration through a 30-µm nylon filter (Sefar Nytal). Harvested conidia were pelleted by centrifugation at 5000×*g* (5 min), resuspended in a 15% (v/v) glycerol solution at a final concentration of 1 × 10^7^ conidia/mL and stored at −80 °C.

For liquid suspension culture, two different carbon sources were included in our studies: glucose (GLU) (Panreac AppliChem), and tomato cell wall (TCW) obtained from tomato (*Lycopersicon esculentum* cv. bola) fruits of commercial maturity and deproteinized as previously reported [10]. In addition, a common culture medium used in botrydial production, modified Czapeck-Dox broth (5% glucose, 0.1% yeast extract, 0.2% NaNO_3_, 0.5% MgSO_4_·7H_2_O, 0.01% FeSO_4_·7H_2_O, 0.5% K_2_HPO_4_), was used. Flasks of 500 mL capacity, containing either 250 mL of modified Czapeck-Dox broth or 250 mL of minimal salt medium (MSM) (50 mM NH_4_Cl, 7.3 mM KH_2_PO_4_, 4.2 mM MgSO_4_, 6.7 mM KCl, 0.07 mM FeSO_4_) supplemented with 1% of the assayed carbon source, were inoculated with three agar plugs (surface = 0.2 × 0.5 cm) taken from the outer edge of 3-day-old colonies of fungal mycelia growing on PDA (Becton Dickinson, Sparks, MD, USA). Cultures were incubated in an orbital shaker at 180 rpm at 22 °C under alternating 12-h light/dark cycles for 5 days. Three independent biological replicates were used per assay. Mycelia were separated by filtration and stored at −80 °C until RNA extraction. The liquid phase was filtered twice through a 30-µm nylon filter (Sefar Nytal) and stored at −80 °C until its use for botrydial determination.

For solid plate culture, three different carbon sources were included in our studies: GLU (Panreac AppliChem), carboxymethyl cellulose (CMC) (Panreac AppliChem) and TCW. For each carbon source, Petri dishes (90 mm) containing MSM Agar medium plus 1% of the assayed carbon source were used. Petri dishes containing malt agar (MA) medium, a common medium used for in vitro solid plate culture of *B. cinerea*, were also used. Plates were inoculated with a 50-µL drop of a *B. cinerea* suspension (5·10^4^ con/mL) placed in the centre of each plate. Twenty independent biological replicates were used per assay. For each assayed carbon source, 10 inoculated Petri dishes were cultivated under 12-h light/dark cycles at 22 °C for 12 days, and 10 were wrapped with aluminium foil and cultivated at 22 °C for the same length of time.

Four different bacteria were used for Agar diffusion tests: *B. subtilis* (CECT 35), *E. faecalis* (CECT 481), *P. fluorescens* (CECT 378) and *S. aureus* (CECT 59). All strains were obtained from the Spanish Type Culture Collection. Isolates were grown on a LB plate and incubated for 24 h at 30 °C. For overnight cultures, one isolate colony was chosen from the plate and incubated in 5 mL of Mueller–Hinton at 180 rpm at 30 °C for 24 h.

### Colony Patterning Depending on the Carbon Source

Mycelial growth rate under all assayed conditions (different carbon sources and light/dark cycles) was calculated from solid plate culture of *B. cinerea* by measurement of the fungal colony diameter, taken as the average length of two diameter measures intersecting at right angles at the centre of the inoculation point. The measurements were made with a millimetre ruler. These measurements were obtained daily for 12 days or until the colony occupied the entire Petri dish. Ten replicates were used for each assayed condition. The mean colony diameter and standard deviation were calculated. The diameters of colonies grown under different conditions were compared, and the effects of the carbon source and light/dark conditions on colony growth were established, with *P* < 0.05 as the threshold of significance, using a generalized linear mixed model (GLMM).

The number of conidia was measured at 4, 6, 8, 10 and 12 days after the inoculation. Spores were collected by gentle mixing with 10 mL of NaCl 0.9% (w/v) on the surface of mature conidiophores and filtration through a 30-µm nylon filter (Sefar Nytal). The conidial suspension obtained was pelleted by centrifugation (5 min, 4000 rpm), resuspended in 1 mL of NaCl 0.9% and gently shaken for homogenization. The spores were counted in a Neubauer count chamber. Five independent replicates were used for each assayed condition. These data were subjected to analysis of variance, with* P* < 0.05 as the threshold of significance.

### Microscopic Morphological Characterization

To study the hyphal branching, for each carbon sourced assayed (GLU, CMC, TCW and MA), several glass slides were covered with a thin layer of MSM Agar plus 1% of the carbon source used or MA, respectively, under sterile conditions. Once the medium had solidified, for each assayed condition, a drop of a conidial suspension of *B. cinerea* 2100 (1 × 10^4^ spores/mL) was spread over the slides and cultivated at 22 °C. At 8, 16 and 48 h post-inoculation, the spores were observed under a light microscope equipped with a digital camera (Motic BA210, Moticam 2.0).

### Analysis of Toxin Production

Unless otherwise noted, materials and reagents were obtained from commercial suppliers and were used without further purification. Chemicals were products of Fluka or Aldrich. All solvents used were freshly distilled. Purification by semipreparative and analytical HPLC was performed with a Hitachi/Merck L-6270 apparatus equipped with a differential refractometer detector (RI-7490). Columns used in the isolation experiments were a LiChrospher^®^ Si 60 (5 µm) LiChroCART^®^ (250 mm × 4 mm) and a LiChrospher^®^ Si 60 (10 µm) LiChroCART^®^ (250 mm × 10 mm). Silica gel (Merck) was used for column chromatography (CC). Thin-Layer Chromatography (TLC) was performed on Merck Kieselgel silica gel 60 F_254_ 0.25-mm-thick glass plates, and Merck RP-18F254, 0.2-mm aluminium silica sheets. ^1^H and ^13^C nuclear magnetic resonance (NMR) measurements were recorded on Varian Unity 400 MHz, Agilent 500 MHz and Varian Inova 600 MHz spectrometers, with SiMe_4_ as the internal reference. Chemical shifts are reported in parts per million (ppm) and were referenced to CDCl_3_ (*δ*_H_ 7.25, *δ*_C_ 77.0). High-resolution mass spectroscopy (HRMS) was recorded with a QTOF mass spectrometer in negative ion electrospray mode at 20 V cone voltage.

For each assayed carbon source, the analysis of toxin production was performed using 250 mL of filtered liquid phase obtained from the *Botrytis* suspension culture after 5 days. Organic compounds were extracted with ethyl acetate (3 × 0.5 mL vol.). The organic extracts were dried over Na_2_SO_4_ and concentrated to dryness, at reduced pressure. This yielded a crude extract (156, 49 and 25 mg of Czapeck-Dox modified, MSM + GLU and MSM + TCW filtered liquid phase, respectively), as a yellow/red oil, which was studied by TLC and column chromatography (CC). Organic extracts were diluted in ethyl acetate, applied over TLC plates using capillary tubes and developed in a TLC chamber with the ethyl acetate/hexane (40:60) solvent system. The developed TLC plates were air-dried and stained with vanillin. Highest purity botrydial and dihydrobotrydial toxins previously isolated from strains of *B. cinerea* were used as the control standard [5]. The crude extract was separated by means of CC on silica gel, with an increasing gradient of ethyl acetate in hexane as a solvent. Extensive spectroscopic methods, specifically ^1^H-NMR and ^13^C-NMR, were then employed to analyse the toxins present in each fraction obtained. The toxin structures were analysed by spectroscopic methods and their structures compared with authentic samples previously isolated from *B. cinerea*.

UPLC-HRESIMS analysis involved a prior treatment of the ethyl acetate extracts by column chromatography on reversed phase C-18 silica (Merck LiChroprep RP-18, 40–63 µm), eluted with methanol (100%) and acetonitrile (100%), yielding two fractions. The acetonitrile fraction yields were 109.8, 38.0 and 19.0 mg for modified Czapeck-Dox, MSM + GLU and MSM + TCW ethyl acetate extracts, whereas the methanol fraction yielded 31.7, 10.3 and 4.2 mg for the modified Czapeck-Dox, MSM + GLU and MSM + TCW ethyl acetate extracts, respectively. Both fractions were collected and analysed in a UPLC-HRESIMS equipment. The methanol fraction did not contain any identifiable compounds.

### Liquid Chromatography–Mass Spectrometry

A Waters Acquity ultra-performance liquid chromatographic (UPLC) system (Waters Corporation, Milford, MA, USA) with an autosampler, a vacuum degasser and a column oven was used. The analytical column used was a Waters ACQUITY UPLC^®^ BEH C18 (2.1 × 50 mm, 1.7 µm, Waters Corporation, Milford, MA, USA). The eluents were water (A) and acetonitrile (B). An initial isocratic elution for 0.5 min (65% A, 35% B), followed by a linear gradient elution from 35 to 100% B in 3 min, was employed, followed by 0.8 min isocratic elution with 100% B, linear gradient elution from 100 to 35% B in 0.7 min and column equilibration for 0.5 min with initial conditions. The flow rate was 0.6 mL/min and the column oven temperature was 40 °C. The flow was directed to a mass spectrometer (MS). UPLC/TOF-HRMS data were acquired with a Waters Xevo QTOF G2 time-of-flight (TOF) high-resolution mass spectrometer (Waters Corporation, Milford, MA, USA) using negative (ESI-) ionization polarity. Leucine enkephalin was used as a lock mass compound ([M-H]^−^ = *m/z* 554.2615). A capillary voltage of 3.5 kV was used, sampling cone voltage 30V, and source temperature was 120 °C. Cone voltage was set at 20V, so molecular ion [M-H]^−^ (C_17_H_25_O_5_, calc. *m/z* 309.1702) was observed for botrydial (C_17_H_26_O_5_). Dihydrobotrydial (C_17_H_28_O_5_) showed the molecular ion [M-H]^−^ (C_17_H_27_O_5_, calc. *m/z* 311.1858) under these experimental conditions. The mass range of *m/z* 180–600 was acquired. A *m/z* window of ± 0.006 amu was used for generating the extracted ion chromatograms (XICs). In the given experimental conditions, detection of botrydial would yield a chromatographic peak in a retention time window of between 1.35 and 1.50 min in any XIC for *m/z* 309.1702, and detection of dihydrobotrydial would yield a chromatographic peak in a retention time window of between 1.20 and 1.35 min in any XIC for *m/z* 311.1858, within the given *m/z* window.

Concentration levels of pure botrydial in calibration solutions were 1, 10 and 100 µg/mL and each of them was injected in duplicate. A calibration curve for botrydial was generated by plotting the peak areas at given concentrations as a function of concentration. The [M-H]^−^ ion was used. A *m/z* window of ± 0.006 amu was used for generating the XICs for botrydial in standards and samples. Solutions of 5 mg/mL (TCW) or 2.5 mg/mL (GLU and CZA) were used for the analysis of botrydial levels in acetonitrile fractions of ethyl acetate fungal extracts.

### Antimicrobial Activity Test

The bactericidal activity of the organic extracts obtained was tested against 4 different bacteria, (*B. subtilis, E. faecalis, P. fluorescens* and *S. aureus*) using the Agar diffusion test. For each assayed bacterium, two Petri dishes (120 mm) containing Mueller–Hinton Agar (Panreac AppliChem) were streaked with 100 µL of an overnight bacterial broth culture to cover the entire surface. Once the surface of the inoculated plate had dried, for each of the organic extracts, 3 wafers (6 mm Ø) containing 1 mg of the organic extracts dissolved in ethyl acetate were placed at equidistant points over the bacterial lawn. Wafers containing only ethyl acetate were placed as controls. Plates were incubated at 30 °C for 18 h and then the diameter of the inhibition zone was observed.

### Isolation of Total RNA, cDNA Synthesis and Quantitative Real-Time PCR

For each condition assayed, 100 mg of frozen mycelia obtained from liquid cultures of *B. cinerea* were individually ground under liquid nitrogen. Powdered mycelia were resuspended in 1 mL of Trizol^®^; RNA isolation was carried out following the manufacturer’s instructions (Invitrogen) but adding a prior stage of homogenization using Fast-Prep^®^-24 (MP Biomedicals): 3 cycles of 60 s at 5.5 m/s, with 450 mg of glass beads (Sigma-Aldrich), and two 1/4 Ceramic Spheres (Q-Biogene) with 1 min on ice between cycles.

Samples of RNA were purified using RQ1 RNase-Free Dnase (Promega) according to the manufacturer’s protocols. The RNA purity and integrity were determined in a NanoDrop spectrophotometer (NanoDrop Technologies). Gel electrophoresis was also performed to verify intact RNA (data not shown). For quantitative RT-PCR analyses, 1 μg of RNA of each sample was reverse-transcribed to cDNA using iScript reverse transcription supermix for RT-qPCR (Bio-Rad), according to the standard protocol. A control reaction was performed without reverse transcriptase for all the isolates, to verify the absence of genomic DNA contamination. Primer pairs were designed to amplify the individual *BcBOT2* gene (NCBI accession number AY277723.2) and the constitutively expressed elongation factor gene EF1b, as the two most stable candidates for normalized *BcBOT2* gene expression (Supplementary Table S1). Quantitative RT-PCR was performed using the CFX 96 Touch™ Real-Time PCR Detection System (Bio-Rad), and the amplifications were performed using the iQ™ SYBR^®^ Green Supermix (Bio-Rad), following the manufacturer’s instructions. PCR cycling parameters were 95 °C for 30 s, followed by 40 cycles at 95 °C for 5 s, 55.7 °C for 30 s. Standard curves were constructed for the selected and endogenous genes from serial dilution of DNA samples. For each experiment, a melting curve analysis was performed to confirm the specificity of the product. Mean Normalized Expression of the selected genes was statistically analysed using the methods of Gil-Salas et al. [15]. Three independent biological replicates were used per assayed carbon source. Three internal replicates were used for each of these biological replicates.

## Results

### Colony Patterning Modifications Depend on the Carbon Source Used

**Fig. 1:**
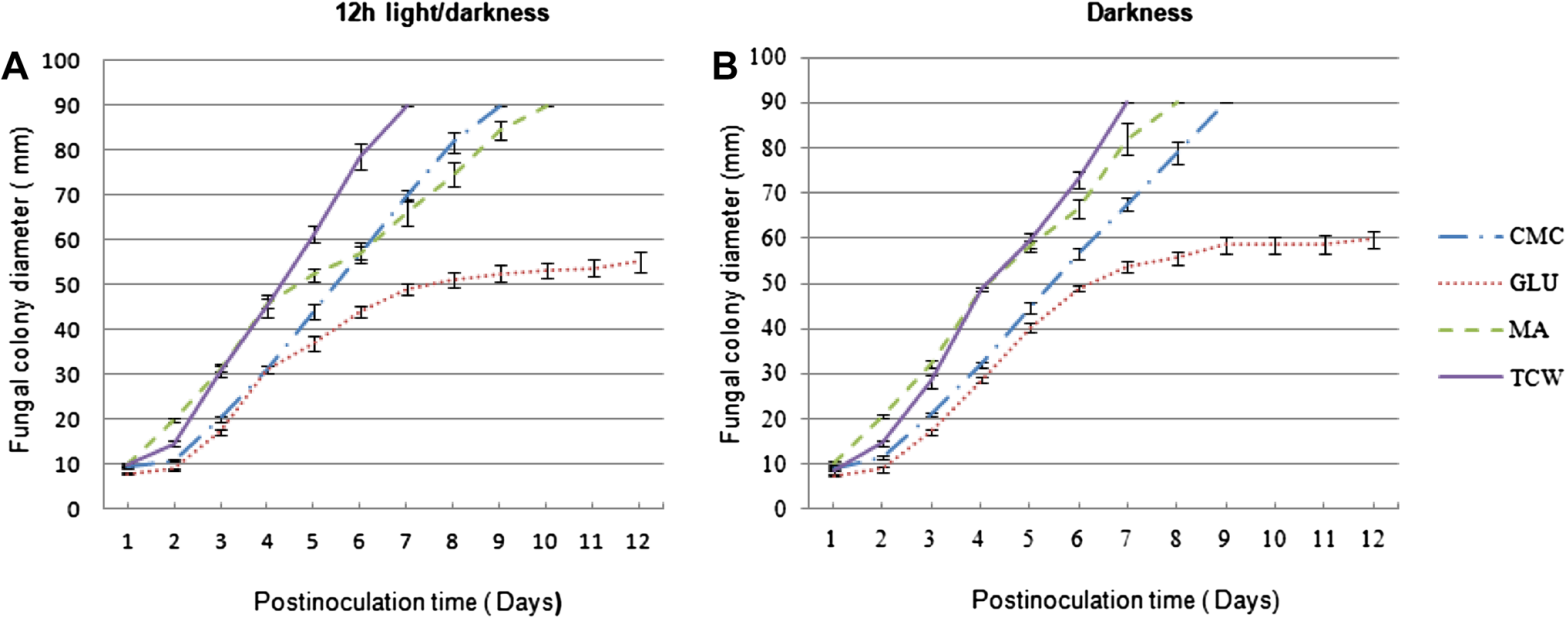
Diameter of growing fungal colony depends on the carbon source. Average fungal colony diameter of *B. cinerea* colony (in mm) plotted against post-inoculation time for *B. cinerea* growing on different carbon sources [glucose (GLU), cellulose (CMC), malt agar medium (MA) and tomato cell wall (TCW)] and under different light/dark conditions: **a** 12-h light/darkness cycles and **b** continuous darkness

**Fig. 2:**
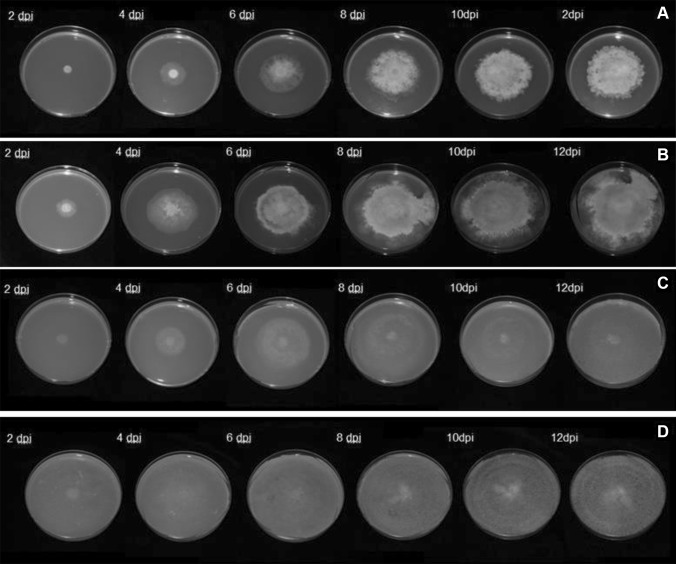
Patterning of fungal colony depends on the carbon source (12-h light/dark cycle). Colony patterning culture of *B. cinerea* 2100 at 22 °C under 12-h light/darkness cycle on **a** minimum salt medium (MSM) plus 1% of GLU as a sole carbon source (MSM + GLU); **b** malt agar culture medium (MA); **c** MSM plus 1% of CMC as a sole carbon source (MSM + CMC) and **d** MSM plus 1% of TCW as a sole carbon source (MSM + TCW)

The influence of different carbon sources on various colony parameters of the *B. cinerea* phenotype, such as mycelial growth rate, mycelial density, shape, sporulation and production of pigments, was examined during a period of 12 days, using three different carbon sources: GLU, CMC and TCW. A control with MA medium was also included. The mycelial growth rate of *B. cinerea* on the four different culture media (MSM-GLU, MSM-CMC, MSM-TCW and MA) and under two different illumination conditions (12-h light/darkness and continuous darkness) was measured daily or until the colony reached the edge of the Petri dish. According to the data collected (Supplementary table S2), *B. cinerea* showed a differential colony growth depending on the available carbon source (Fig. 1). The influence of the carbon source on colony diameter was statistically proven by a GLMM analysis of the data with a confidence level of 95% (*P* > 0.05). For both lighting conditions of culture, the slowest mycelial growth was exhibited by Botrytis on MSM Agar plus 1% GLU as a sole carbon source, which reached the smallest colony diameter measured, with a large area of the plate left unoccupied after 12 days; however, with the rest of the carbon sources assayed, the growth of the fungus continued until it reached the edge of the Petri dish. On the other hand, the fastest mycelial growth rate was recorded for the TCW-supplemented cultures, which gained colony size rapidly, reaching the edge of the plate at 7 days post-inoculation (dpi) (Fig. 1). With respect to the light/darkness culture conditions assayed, statistically significant differences in mycelial growth rate were shown only for Botrytis grown on MA and MSM + GLU (Supplementary Fig. S2). Mycelial growth rate is higher in continuous darkness, from the fourth dpi, on MA medium, and from the sixth dpi on the MSM + GLU medium. In all the conditions assayed, differences in mycelial density, colony shape and production of pigment related to the carbon source were detected in the fungal plate cultures (Fig. 2 and Supplementary Fig. S1). The hyphal layer was most dense when *B. cinerea* was cultured on MSM + GLU and MA, resulting in fluffy radial colonies which exhibited roughened or ramified shapes and intense red reverse colour. On the other hand, on MSM + CMC and MSM + TCW culture media, colonies with a thin mycelial layer were observed. A weak pigment production was observed on MSM + TCW, whereas there was no pigment production when the fungus was cultured on MSM + CMC.

With respect to conidial production, the concentration of conidia obtained was measured at 4, 6, 8, 10 and 12 days post-inoculation. The highest level of conidial production was recorded on the media supplemented with TCW, under both conditions of light and darkness assayed. The production of conidia in MA and CMC was similar: in both cases, much higher values were presented compared with GLU. The influence of light/darkness incubations was also clear: higher conidial concentrations were obtained at 12 dpi in all the plates under the 12-h light/dark condition. However, with GLU the graph presents an anomalous profile, with conidial production under the 12-h light condition remaining very low until the final days of the assay (Supplementary Fig. S3).

### Microscopic Morphological Characterization

**Fig. 3:**
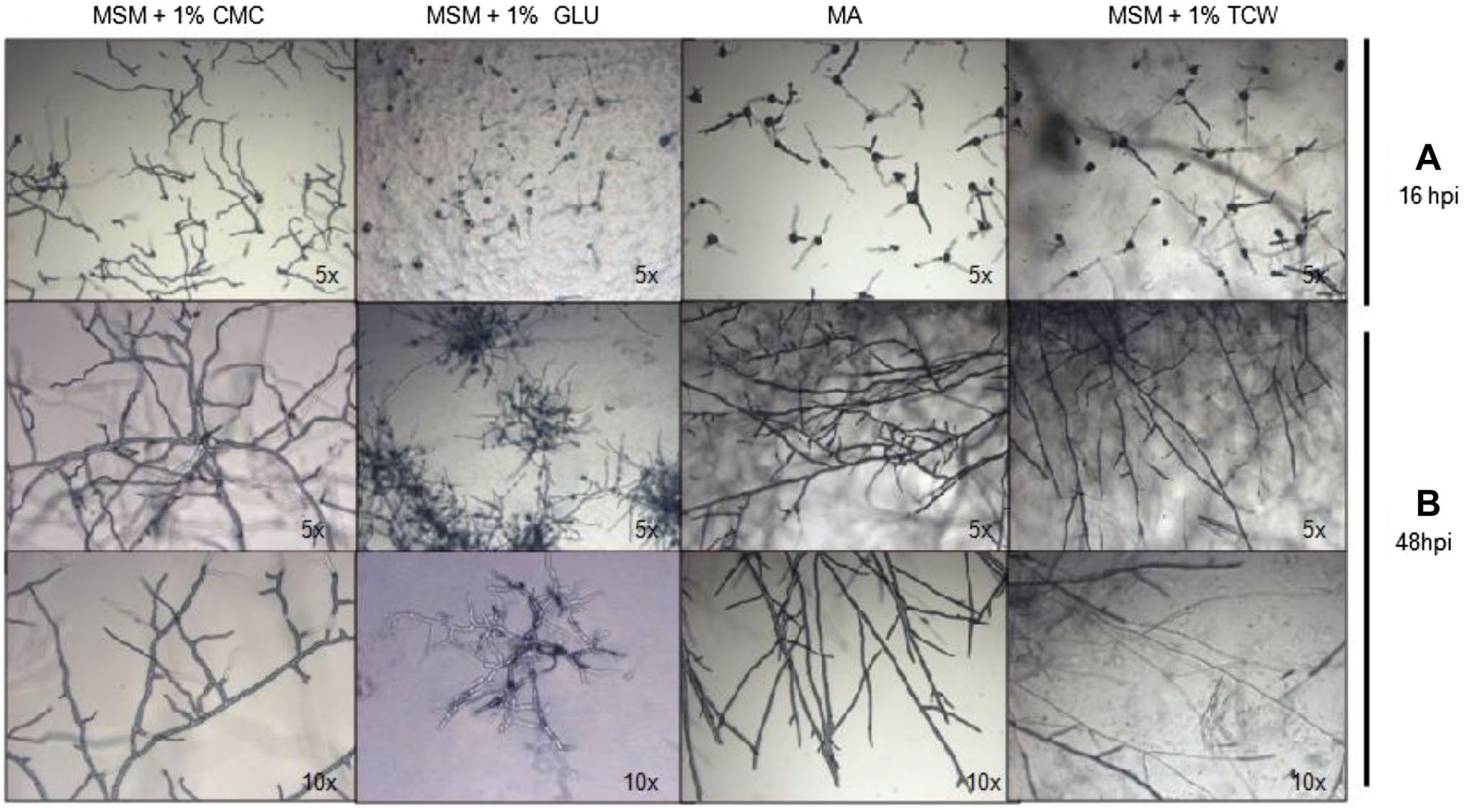
Microscopic morphological characterization. Observation, under light microscopy, of mycelia of *B. cinerea* 2100 grown on culture media with different available carbon sources at **a** 16 and **b** 48 h post-inoculation (hpi)

Microscopy was used to follow mycelial formation in the presence of different carbon sources. By 8 h post-inoculation (hpi), germination of the conidia had occurred in all conditions assayed. Almost 100% of the conidia had a visible germ tube (data not shown). By 16 hpi, in the presence of MSM + GLU, the fungus showed short and unbranched hyphae that, by 48 hpi, resulted in a compact mycelium formed by hyphae of short length and with reduced space between branches (Fig. 3). In MSM + CMC, by 16 hpi, the conidia germinated had formed long branching hyphae that evolved into thin mycelia of long hyphae with large spaces between branches (Fig. 3). With the other two carbon sources assayed, MA (control) and MSM + TCW, the result by 48 hpi was mycelia with long hyphae, and there were noticeably fewer branches with TCW as a carbon source (Fig. 3).

### Analysis of Toxin Production: Botrydial and Dihydrobotrydial

The influence of carbon source on the production of phytotoxins was evaluated by identification of botrydial, a characteristic virulence factor of *B. cinerea* [15] and of dihydrobotrydial, its metabolic sink [9] in culture filtrates from *B. cinerea* grown individually in MSM plus 1% of a single carbon source (TCW or glucose) and in Czapeck-Dox modified medium. As a first approach, organic extracts from all assayed conditions were studied by TLC and column chromatography. Data clearly showed the presence of botrydial and dihydrobotrydial in extracts from *Botrytis* cultured in MSM + GLU and Czapeck-Dox modified medium. However, organic extracts from *Botrytis* cultured in MSM + TCW did not show the presence of these phytotoxins (Supplementary Fig. S4).

In order to increase the sensitivity of the detection method and validate the results, fungal extracts were analysed by ^1^H-NMR and ULPC-HRESIMS. First, the crude extracts were separated by means of column chromatography on silica gel, with an increasing gradient of ethyl acetate in hexane. Final purification by HPLC of the fractions obtained from fermentations on MSM + GLU and Czapeck-Dox modified medium (CZA) yielded, according to TLC results, two compounds, the ^1^H- and ^13^C-NMR spectra (Supplementary Fig. S5) of which were identical to those of botrydial and dihydrobotrydial [5]. However, these toxins were not detected in the extract obtained by the culture using TCW as a carbon source.

For the UPLC-HRESIMS analysis, the acetonitrile fraction of the ethyl acetate extract of each fungal culture (GLU, CZA and TCW), obtained as described above, was analysed by UPLC coupled to HRESIMS. For each fungal culture, total ion current (TIC) chromatograms were obtained, which were further processed to obtain the extracted ion chromatograms (XICs) corresponding to the characteristic molecular ions for botrydial ([M-H]^−^, *m/z* 309.1702) and dihydrobotrydial ([M-H]^−^, *m/z* 311.1858) with a mass window of 0.006 uma (Supplementary Fig. S6).

Both CZA and GLU fungal extracts showed common chromatographic patterns in their XICs, where dihydrobotrydial was found within a retention time window of between 1.20 and 1.35 min, and botrydial was found within a retention time window of between 1.35 and 1.50 min (Supplementary Fig. S6). In contrast, neither dihydrobotrydial nor botrydial chromatographic peaks due to their characteristic molecular ions ([M-H]^−^) were observed for the TCW fungal extract within their respective retention time windows reported above. This observation further supports the previously described TLC and ^1^H-NMR findings.

A semi-quantitative determination of botrydial was made for the acetonitrile fraction of ethyl acetate extracts (for CZA, GLU and TCW). According to this, botrydial content in GLU and CZA was, respectively, 44.3 micrograms and 33.6 micrograms per mg of each acetonitrile fraction. The lower concentration used for the evaluation of the botrydial level with the UPLC-HRESIMS method described above was 1 µg/mL. Such a concentration would be equivalent to a botrydial level of 0.2 µg/mg in the TCW acetonitrile fraction of the ethyl acetate extract used for this study. Therefore, under the given experimental conditions, if botrydial had been present in TCW extracts, it should be at a level below the 0.2 µg/mg level mentioned above. This is a much lower level of botrydial than that observed for the CZA and GLU extracts.

In conclusion, our findings showed the presence of botrydial and dihydrobotrydial in filtrates from *B. cinerea* cultured in Czapeck-Dox modified medium and in MSM + GLU, but neither of these toxins was detected when TCW was the only carbon source present in the culture medium.

### Antimicrobial Activity Test

In our study, only the organic extract from *B. cinerea* cultured on MSM + GLU and Czapeck-Dox modified medium showed significant antimicrobial activity against all the bacterial strains assayed (see Supplementary Fig. S7).

### Study of Toxin Biosynthesis Gene Expression Depending on the Carbon Source

The *BcBOT2* gene encodes a sesquiterpene cyclase that plays a key role in botrydial biosynthesis [19]. Since it has been identified as a part of the botrydial biosynthetic gene cluster, coding for the pentalenene sesquiterpene cyclase, *BcBOT2* is an excellent candidate for controlling the production of botrydial at expression level. Considering this, and based on the above result, we used quantitative Real-Time PCR (RT-qPCR) to test the possible influence of the available carbon source on Botrydial biosynthesis. Two different carbon sources were assayed: Glucose and TCW; a Czapeck-Dox modified medium was also included in our study as a positive control. Good efficiencies and linear regressions for all the genes are shown in Supplementary Table S1. RT-qPCR results indicated a significant downregulation of *BcBOT2* when TCW is the sole available carbon source (Supplementary Fig. S8).

## Discussion

*Botrytis cinerea* is able to deploy a wide range of strategies to complete its infection cycle. The induction of these strategies must be mainly in response to the environmental conditions, as has been previously described [13]. In previous assays, the growth medium used has been shown to have a significant effect on colony patterning in different filamentous fungi such as *Aspergillus oryzae* [18], *Penicillium* spp., *Acremonium kiliense* and *Fusarium oxysporum* [21], and *B. cinerea* [17]. In the laboratory, in vitro cultures of *B. cinerea* are usually carried out using common culture media such as Potato Dextrose Agar (PDA), Czapeck-Dox or MA. In those media, glucose is usually the main source of carbon. Glucose is the universal energetic unit, it can be assimilated by the vast majority of living organisms and it induces a constitutive response in *B. cinerea*. The negative aspect is that the culture media employed do not only have an effect on colony patterning, but also seem to prevent the induction of other compounds or processes that may be produced by the fungi during the infection process. A previous report has shown that, for example, at the level of the *B. cinerea* secretome, only 5% of the predicted secreted proteins listed in the genome database have been found in proteomics studies [14]. Moreover, the secretome 2DE profile obtained presents a strong dependence on the carbon source used [13]. In secondary metabolism, the situation is the same. Several secondary metabolism genes involved at the genome level are associated with the production of new metabolites; however, those cryptic compounds have not been found in *B. cinerea* liquid cultures, probably because their production is inhibited or suppressed by the culture and environmental conditions.

In our study, with the object of finding new induction mechanisms and evaluating the possible influence of the carbon source and plant-based elicitors over the *B. cinerea* phenotype, MSM plus 1% of a sole carbon source was used as a culture medium. Three different sole carbon sources, GLU, CMC and TCW, were used. The intention was to gradually increase the complexity of the carbon source, starting from glucose, as an easily assimilable carbon source (typically used in “in vitro” culture media), then cellulose, as a major component of plant cell walls [3, 12, 22], and finally TCW as the closest approximation to an “in planta” infection. CMC and TCW have been reported previously to play the role of plant-based elicitors in *B. cinerea* and trigger specific responses in the fungus [12, 13]. MA, a common “in vitro” culture medium, was also included in our study. Our findings are consistent with previous data and show a clear influence of the carbon source on all the phenotypic parameters measured in *B. cinerea* colonies. Data show a directly proportional relationship between the complexity of the assayed carbon source and the fungal growth rate, with a statistical significance of 95% (*P* < 0.05). The growth rate is highest when TCW is the only available carbon source and lowest when glucose is used. On the other hand, mycelial density and production of pigments both decrease with increasing complexity of the carbon source. We assume that, given an easily assimilable nutrient in the media, the fungus has less need to invade nearby plant tissues with a massive hyphal growth, and this behaviour is replicated under in vitro conditions. In spite of the influence that the light/dark cycle has on *B. cinerea* biology, we found that its effect on fungal growth rate is dependent of the carbon source used; in cultures with TCW and CMC as the sole carbon source, there were no significant differences between colonies grown in darkness or light. On the other hand, in cultures with GLU and MA, the differences between colonies grown in darkness or light were statistically significant, and a higher growth rate was presented in the culture condition of darkness. Measuring the production of spores, the data obtained fit nicely with the growth rate. Conidial production is higher in plant-based media (CMC and TCW) than in easily assimilable media rich in carbohydrates (GLU and MA), again independently of the light cycle. Both sets of data support the hypothesis that, when *B. cinerea* can access an easily assimilable carbon source, it has less need to develop the structures for invading the host plant and spreading the infection. The results obtained show clearly that the carbon source used produces phenotypic changes in *B. cinerea;* these changes, therefore, must be produced by the expression of a differential set of genes. The induction of the expression of these cryptic genes may allow researchers to detect and study new virulence factors associated with the pathogenicity methods or secondary metabolism of the fungus.

To progress in this direction, a differential analysis of the production of botrydial was carried out. TCW, used as a sole carbon source, appears to inhibit the production of botrydial and derivatives, as evident from the results obtained by chromatography techniques, ^1^H-NMR experiments and UPLC-HRESIMS. Inhibition in the production of toxins has been supported by repression of the *BcBOT2* gene. Depending on the available carbon source, a differential expression pattern of *BcBOT2* is observed: the gene is expressed in GLU and inhibited in TCW. These results suggest clearly that the fungus adapts to the environment and to the host. Studies are in progress to elucidate the production of potential cryptic metabolites by the expression of genes involved in silent biosynthetic pathways.

Summarizing the data obtained in our research, we have found that variations in the carbon source or plant-based elicitor cause variations in the observed phenotype. Those variations include alterations in the growth rates, hyphal morphology and conidial production. Moreover, we were able to establish that, when TCW is used as a sole carbon source, production of both botrydial and dihydrobotrydial, the main toxins of *B. cinerea*, is inhibited. Expression of the toxin biosynthesis gene, *BcBOT2*, is also repressed. It has previously been reported that when TCW is used as a sole carbon source, the production of cell wall-degrading enzymes (CWDEs) is induced, but not so when GLU is used [13]. These data suggest a possible connection between environmental conditions and the expression of virulence factors. All the foregoing data support the hypothesis of the existence of a time lapse in the expression of the virulence factors. CWDEs are produced to invade plant tissues and suppress external barriers, and when easily assimilable sugars from inner plant tissues are available, the toxin production machinery is turned on. This suggests the presence of a molecular switch that turns on and off the chemical or enzymatic virulence mechanisms of the fungus. The nature of this mechanism still remains to be elucidated.

## Electronic supplementary material

Below is the link to the electronic supplementary material.

Supplementary material 1 (PPTX 1219 KB)
