# Supplementary material for: Phenotypic Effects and Inhibition of Botrydial Biosynthesis Induced by Different Plant-Based Elicitors in Botrytis cinerea
Source: Curr Microbiol. 2017 Nov 17;75(4):431–40. doi: 10.1007/s00284-017-1399-3 (PMC5842495; doi:10.1007/s00284-017-1399-3)
Supplement: Supplementary file 1 — Supplementary material 1 (PPTX 1219 KB) [file 284_2017_1399_MOESM1_ESM.pptx]

## Slide 1
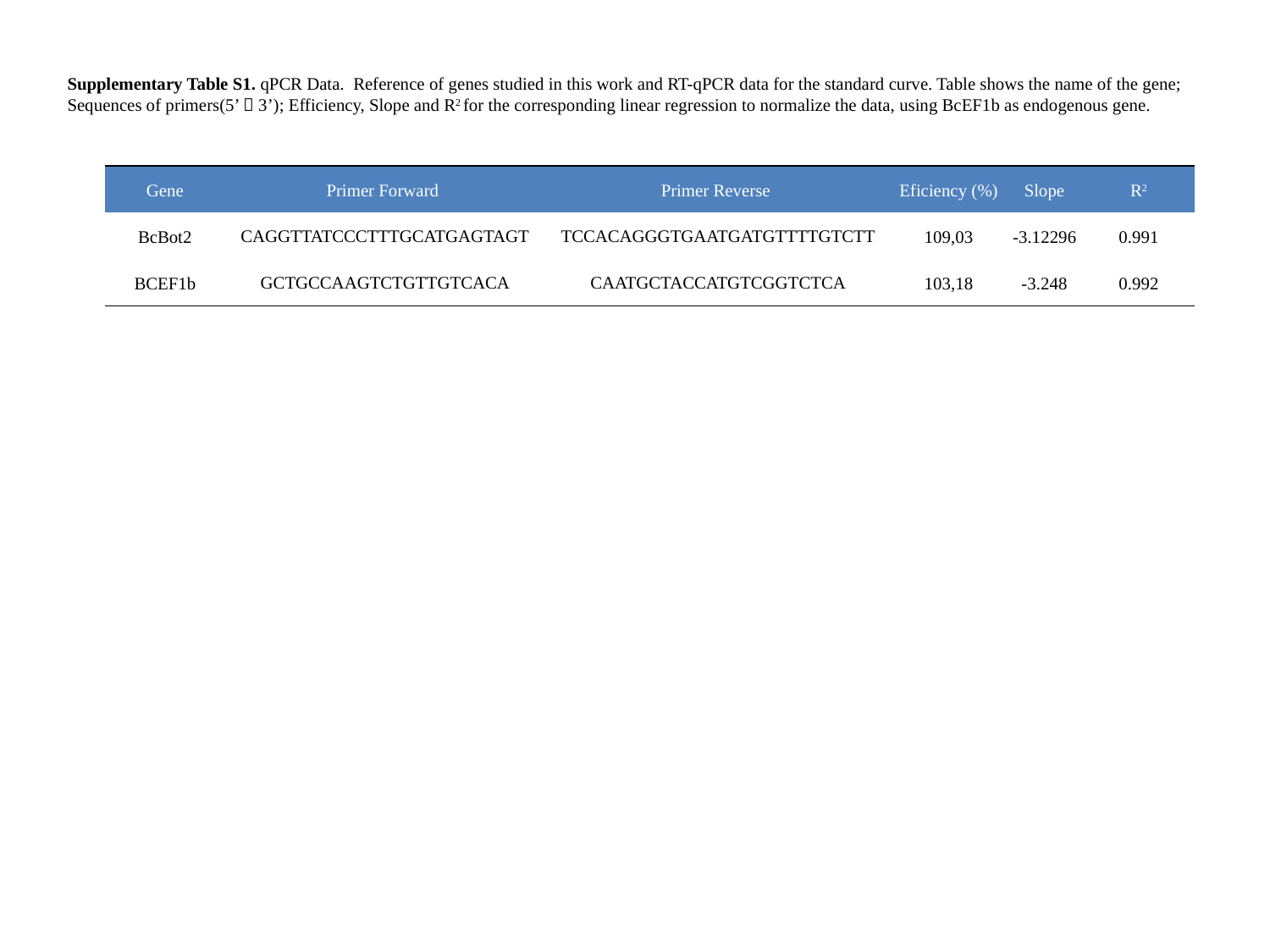

Supplementary Table S1. qPCR Data. Reference of genes studied in this work and RT-qPCR data for the standard curve. Table shows the name of the gene; Sequences of primers(5’  3’); Efficiency, Slope and R2 for the corresponding linear regression to normalize the data, using BcEF1b as endogenous gene.
| Gene | Primer Forward | Primer Reverse | Eficiency (%) | Slope | R2 |
| --- | --- | --- | --- | --- | --- |
| BcBot2 | CAGGTTATCCCTTTGCATGAGTAGT | TCCACAGGGTGAATGATGTTTTGTCTT | 109,03 | -3.12296 | 0.991 |
| BCEF1b | GCTGCCAAGTCTGTTGTCACA | CAATGCTACCATGTCGGTCTCA | 103,18 | -3.248 | 0.992 |

## Slide 2
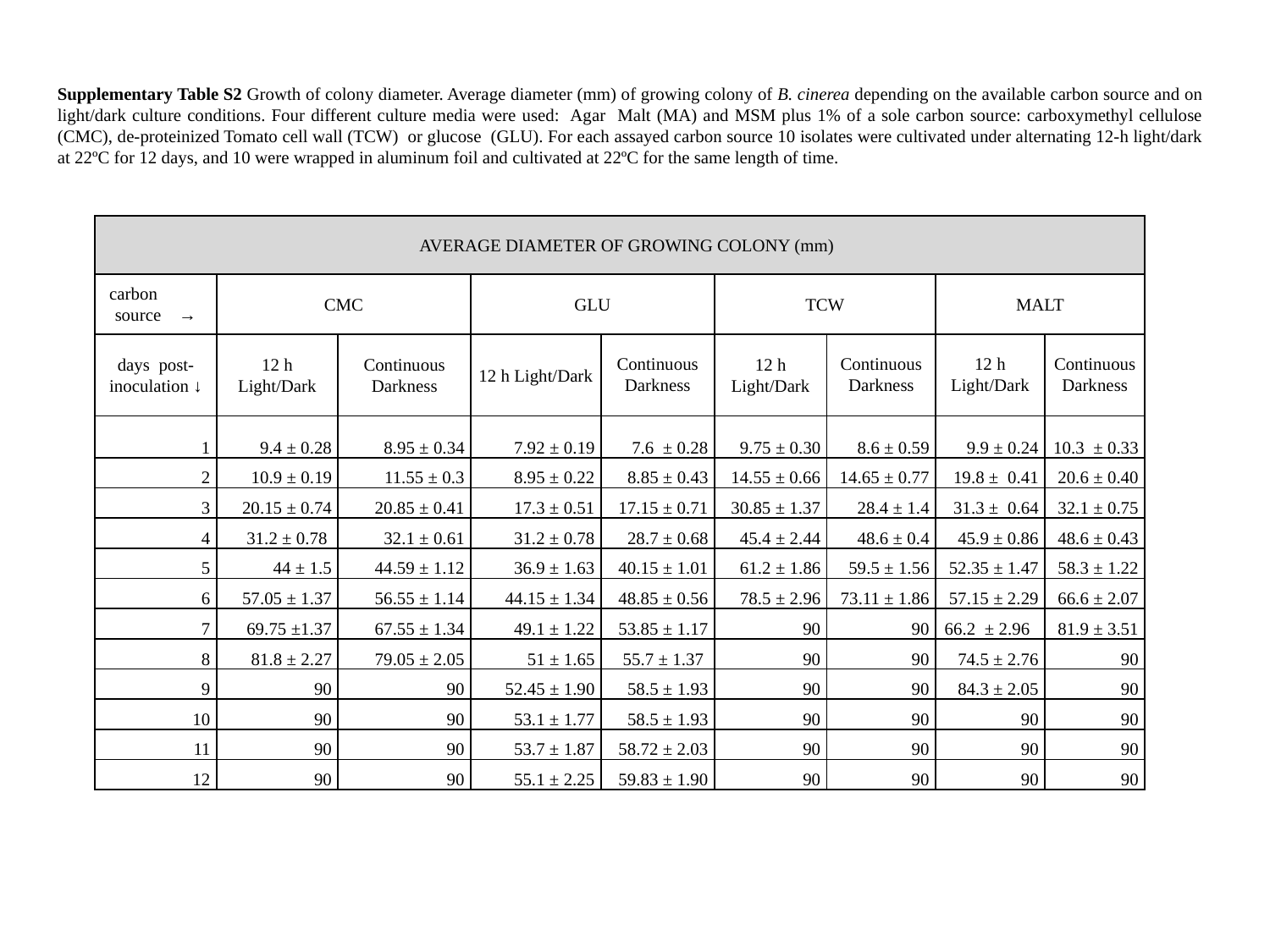

Supplementary Table S2 Growth of colony diameter. Average diameter (mm) of growing colony of B. cinerea depending on the available carbon source and on light/dark culture conditions. Four different culture media were used: Agar Malt (MA) and MSM plus 1% of a sole carbon source: carboxymethyl cellulose (CMC), de-proteinized Tomato cell wall (TCW) or glucose (GLU). For each assayed carbon source 10 isolates were cultivated under alternating 12-h light/dark at 22ºC for 12 days, and 10 were wrapped in aluminum foil and cultivated at 22ºC for the same length of time.
| AVERAGE DIAMETER OF GROWING COLONY (mm) | | | | | | | | |
| --- | --- | --- | --- | --- | --- | --- | --- | --- |
| carbon source → | CMC | | GLU | | TCW | | MALT | |
| days post-inoculation ↓ | 12 h Light/Dark | Continuous Darkness | 12 h Light/Dark | Continuous Darkness | 12 h Light/Dark | Continuous Darkness | 12 h Light/Dark | Continuous Darkness |
| 1 | 9.4 ± 0.28 | 8.95 ± 0.34 | 7.92 ± 0.19 | 7.6 ± 0.28 | 9.75 ± 0.30 | 8.6 ± 0.59 | 9.9 ± 0.24 | 10.3 ± 0.33 |
| 2 | 10.9 ± 0.19 | 11.55 ± 0.3 | 8.95 ± 0.22 | 8.85 ± 0.43 | 14.55 ± 0.66 | 14.65 ± 0.77 | 19.8 ± 0.41 | 20.6 ± 0.40 |
| 3 | 20.15 ± 0.74 | 20.85 ± 0.41 | 17.3 ± 0.51 | 17.15 ± 0.71 | 30.85 ± 1.37 | 28.4 ± 1.4 | 31.3 ± 0.64 | 32.1 ± 0.75 |
| 4 | 31.2 ± 0.78 | 32.1 ± 0.61 | 31.2 ± 0.78 | 28.7 ± 0.68 | 45.4 ± 2.44 | 48.6 ± 0.4 | 45.9 ± 0.86 | 48.6 ± 0.43 |
| 5 | 44 ± 1.5 | 44.59 ± 1.12 | 36.9 ± 1.63 | 40.15 ± 1.01 | 61.2 ± 1.86 | 59.5 ± 1.56 | 52.35 ± 1.47 | 58.3 ± 1.22 |
| 6 | 57.05 ± 1.37 | 56.55 ± 1.14 | 44.15 ± 1.34 | 48.85 ± 0.56 | 78.5 ± 2.96 | 73.11 ± 1.86 | 57.15 ± 2.29 | 66.6 ± 2.07 |
| 7 | 69.75 ±1.37 | 67.55 ± 1.34 | 49.1 ± 1.22 | 53.85 ± 1.17 | 90 | 90 | 66.2 ± 2.96 | 81.9 ± 3.51 |
| 8 | 81.8 ± 2.27 | 79.05 ± 2.05 | 51 ± 1.65 | 55.7 ± 1.37 | 90 | 90 | 74.5 ± 2.76 | 90 |
| 9 | 90 | 90 | 52.45 ± 1.90 | 58.5 ± 1.93 | 90 | 90 | 84.3 ± 2.05 | 90 |
| 10 | 90 | 90 | 53.1 ± 1.77 | 58.5 ± 1.93 | 90 | 90 | 90 | 90 |
| 11 | 90 | 90 | 53.7 ± 1.87 | 58.72 ± 2.03 | 90 | 90 | 90 | 90 |
| 12 | 90 | 90 | 55.1 ± 2.25 | 59.83 ± 1.90 | 90 | 90 | 90 | 90 |

## Slide 3
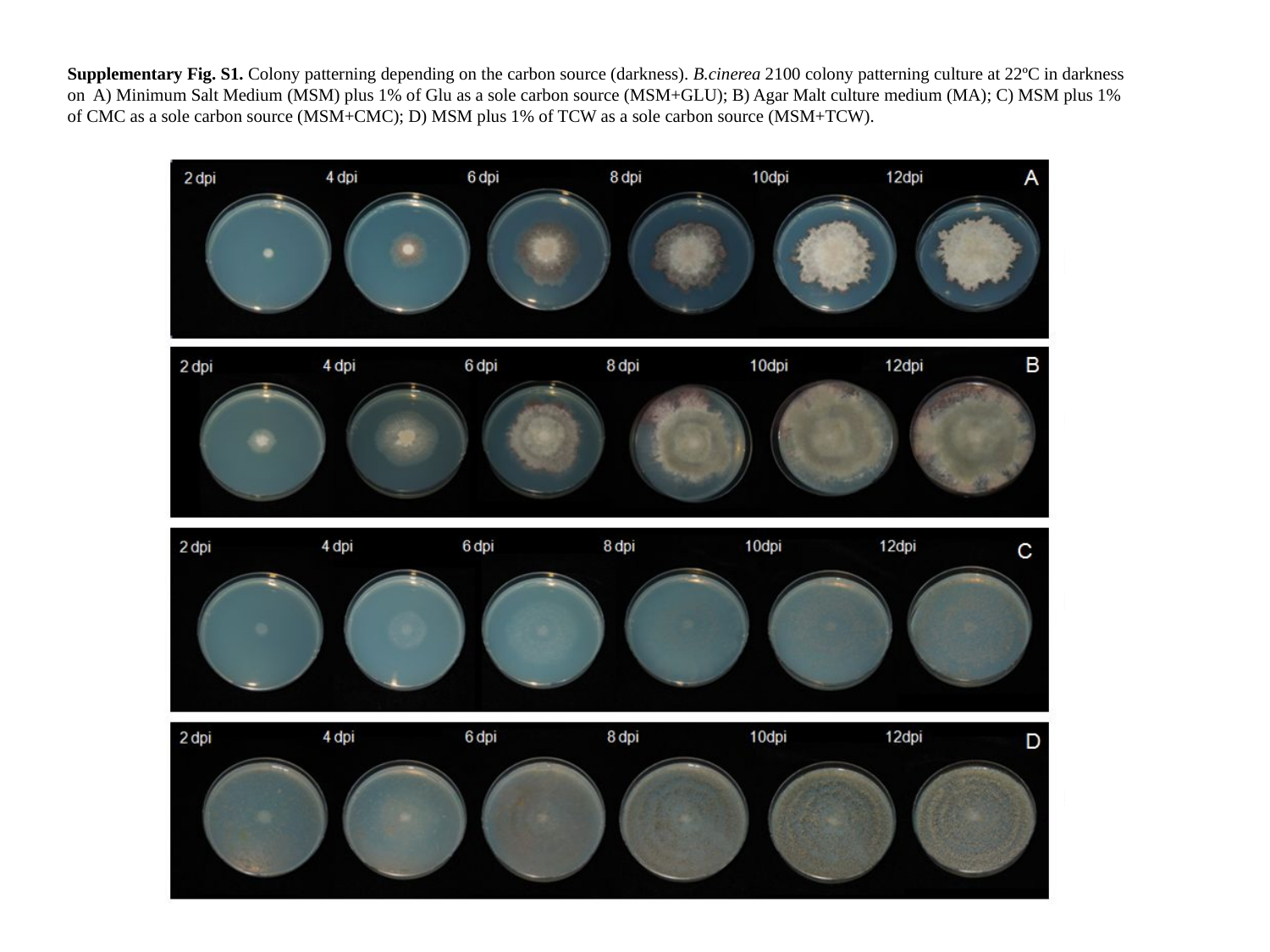

Supplementary Fig. S1. Colony patterning depending on the carbon source (darkness). B.cinerea 2100 colony patterning culture at 22ºC in darkness on A) Minimum Salt Medium (MSM) plus 1% of Glu as a sole carbon source (MSM+GLU); B) Agar Malt culture medium (MA); C) MSM plus 1% of CMC as a sole carbon source (MSM+CMC); D) MSM plus 1% of TCW as a sole carbon source (MSM+TCW).

## Slide 4
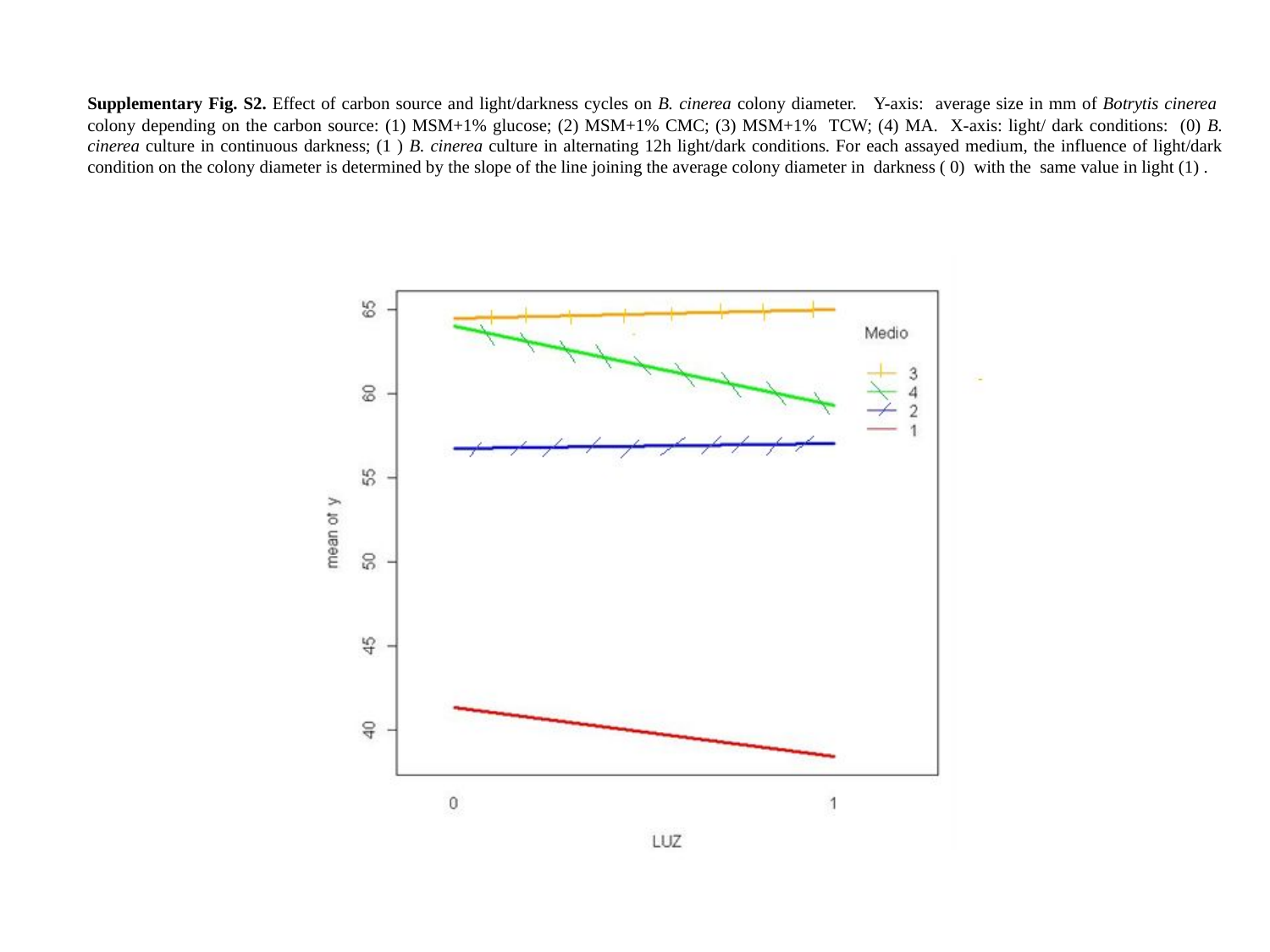

Supplementary Fig. S2. Effect of carbon source and light/darkness cycles on B. cinerea colony diameter. Y-axis: average size in mm of Botrytis cinerea colony depending on the carbon source: (1) MSM+1% glucose; (2) MSM+1% CMC; (3) MSM+1% TCW; (4) MA. X-axis: light/ dark conditions: (0) B. cinerea culture in continuous darkness; (1 ) B. cinerea culture in alternating 12h light/dark conditions. For each assayed medium, the influence of light/dark condition on the colony diameter is determined by the slope of the line joining the average colony diameter in darkness ( 0) with the same value in light (1) .

## Slide 5
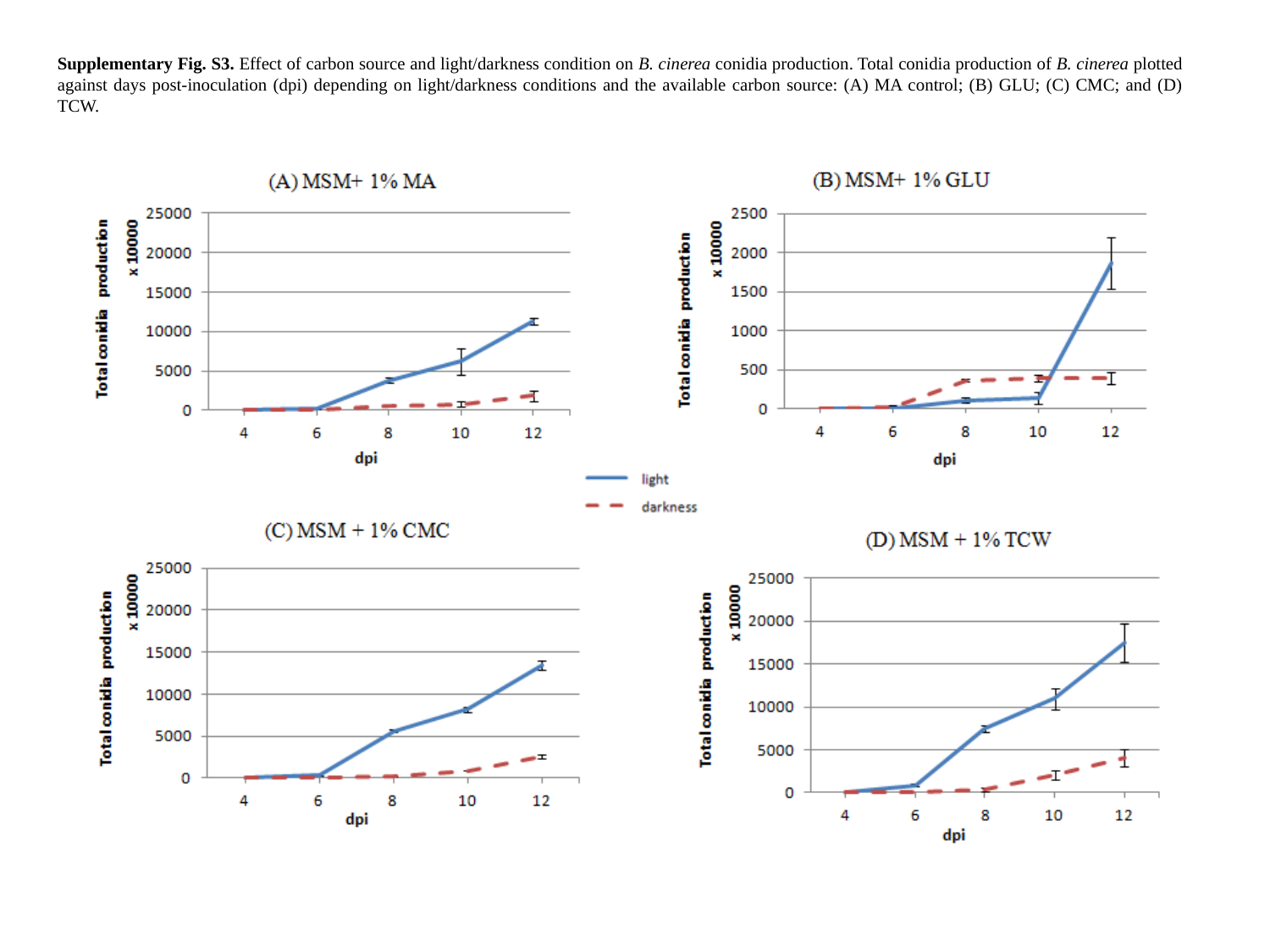

Supplementary Fig. S3. Effect of carbon source and light/darkness condition on B. cinerea conidia production. Total conidia production of B. cinerea plotted against days post-inoculation (dpi) depending on light/darkness conditions and the available carbon source: (A) MA control; (B) GLU; (C) CMC; and (D) TCW.

## Slide 6
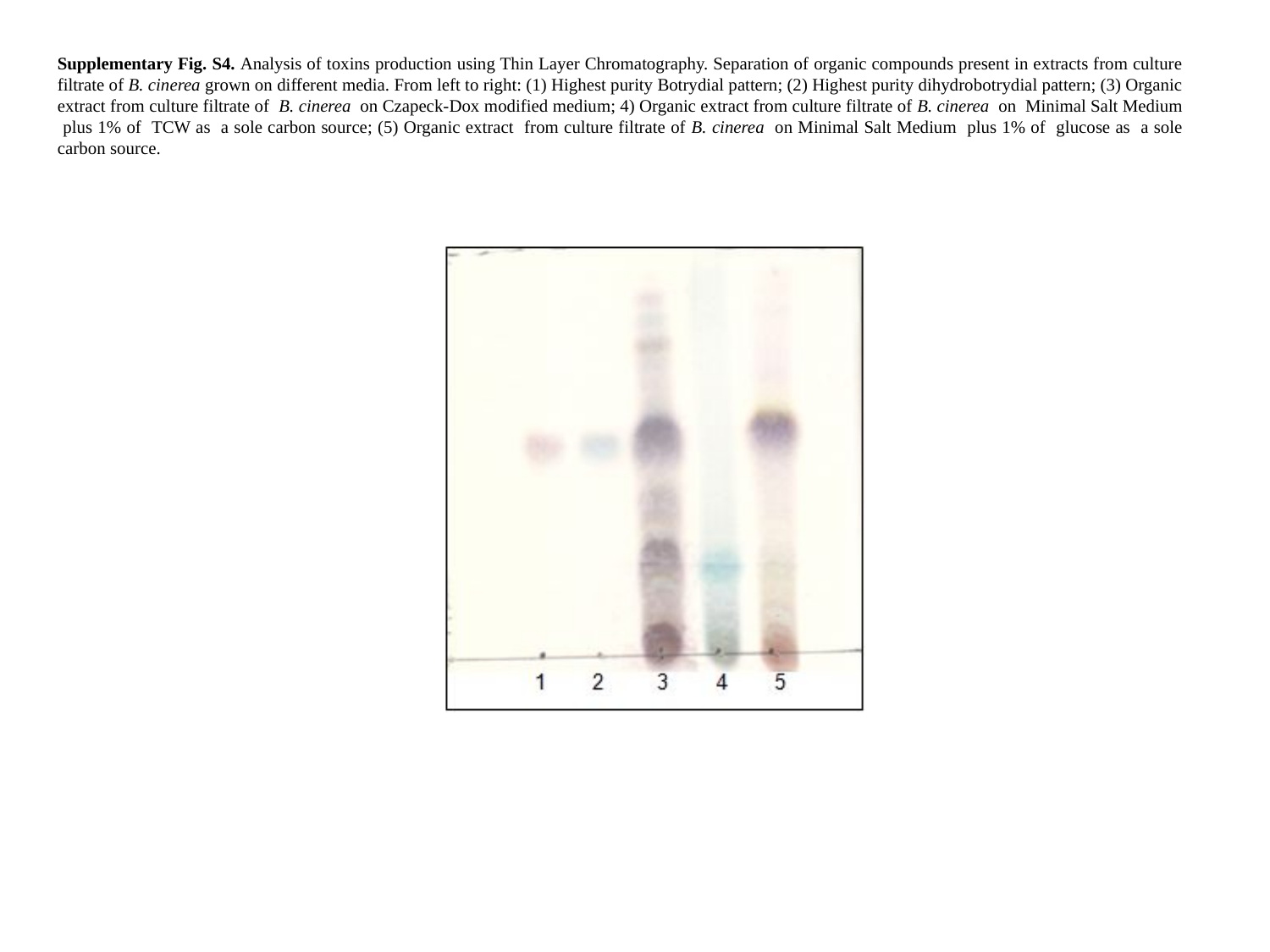

Supplementary Fig. S4. Analysis of toxins production using Thin Layer Chromatography. Separation of organic compounds present in extracts from culture filtrate of B. cinerea grown on different media. From left to right: (1) Highest purity Botrydial pattern; (2) Highest purity dihydrobotrydial pattern; (3) Organic extract from culture filtrate of B. cinerea on Czapeck-Dox modified medium; 4) Organic extract from culture filtrate of B. cinerea on Minimal Salt Medium plus 1% of TCW as a sole carbon source; (5) Organic extract from culture filtrate of B. cinerea on Minimal Salt Medium plus 1% of glucose as a sole carbon source.

## Slide 7
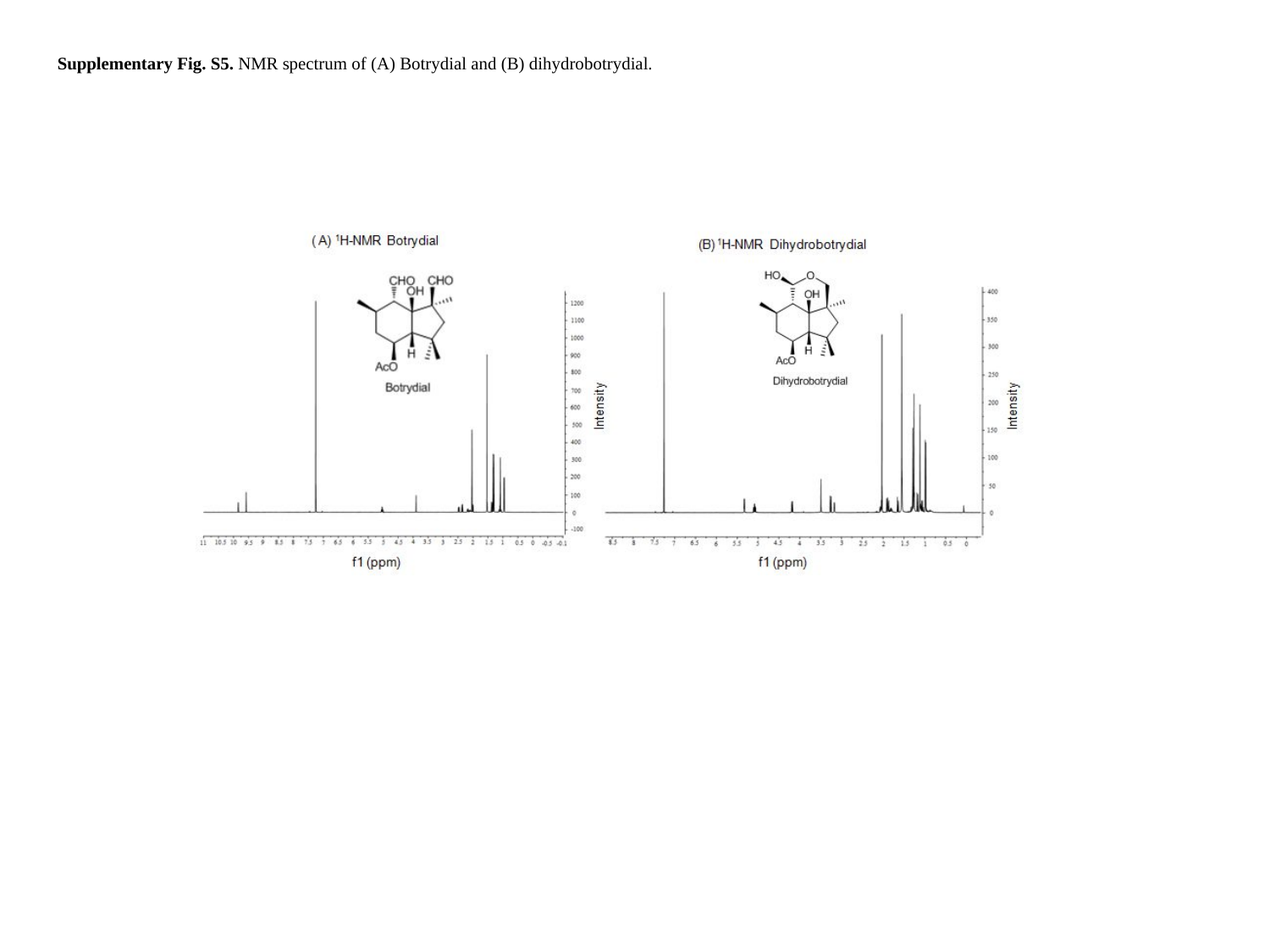

Supplementary Fig. S5. NMR spectrum of (A) Botrydial and (B) dihydrobotrydial.

## Slide 8
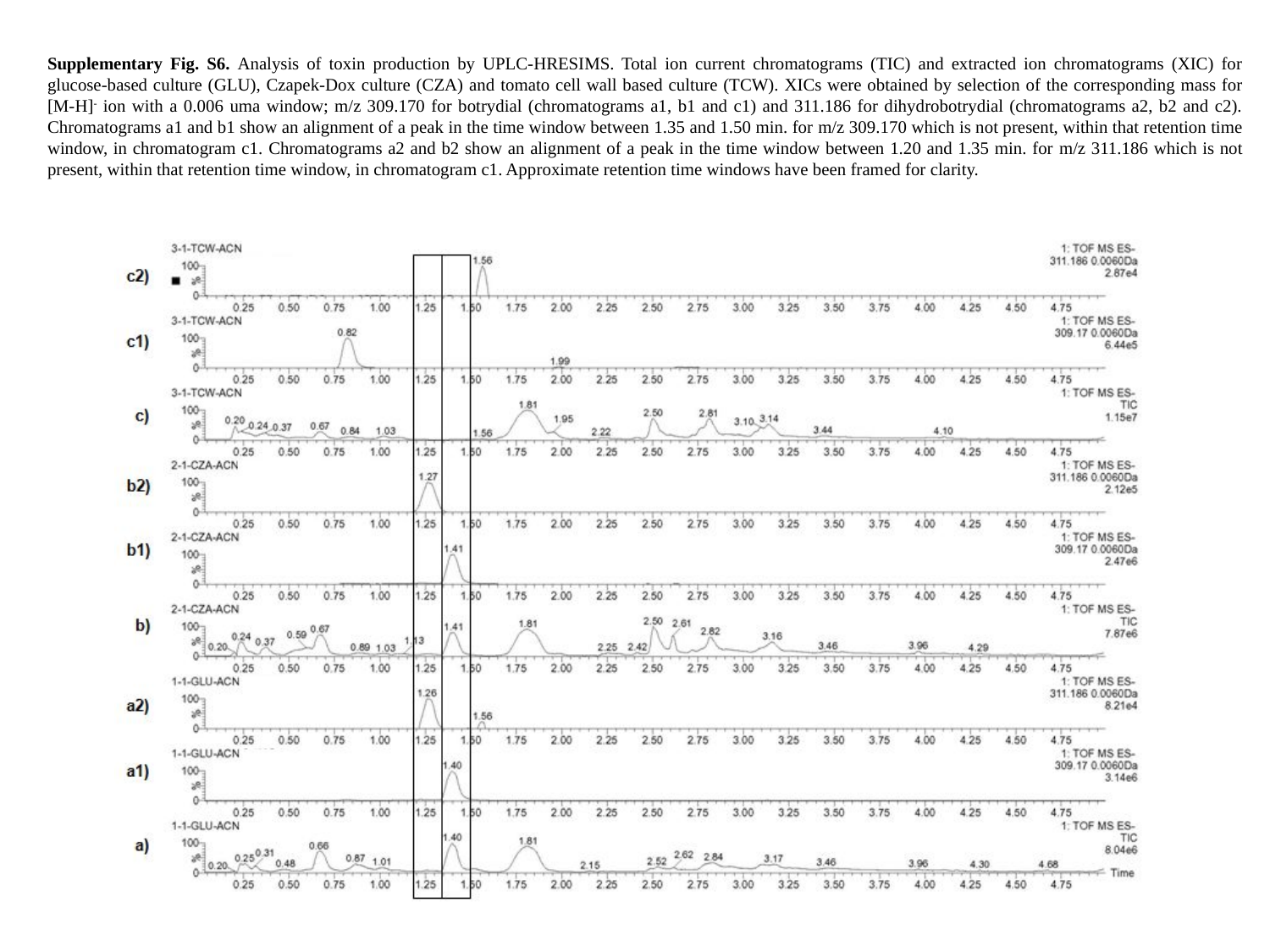

Supplementary Fig. S6. Analysis of toxin production by UPLC-HRESIMS. Total ion current chromatograms (TIC) and extracted ion chromatograms (XIC) for glucose-based culture (GLU), Czapek-Dox culture (CZA) and tomato cell wall based culture (TCW). XICs were obtained by selection of the corresponding mass for [M-H]- ion with a 0.006 uma window; m/z 309.170 for botrydial (chromatograms a1, b1 and c1) and 311.186 for dihydrobotrydial (chromatograms a2, b2 and c2). Chromatograms a1 and b1 show an alignment of a peak in the time window between 1.35 and 1.50 min. for m/z 309.170 which is not present, within that retention time window, in chromatogram c1. Chromatograms a2 and b2 show an alignment of a peak in the time window between 1.20 and 1.35 min. for m/z 311.186 which is not present, within that retention time window, in chromatogram c1. Approximate retention time windows have been framed for clarity.

## Slide 9
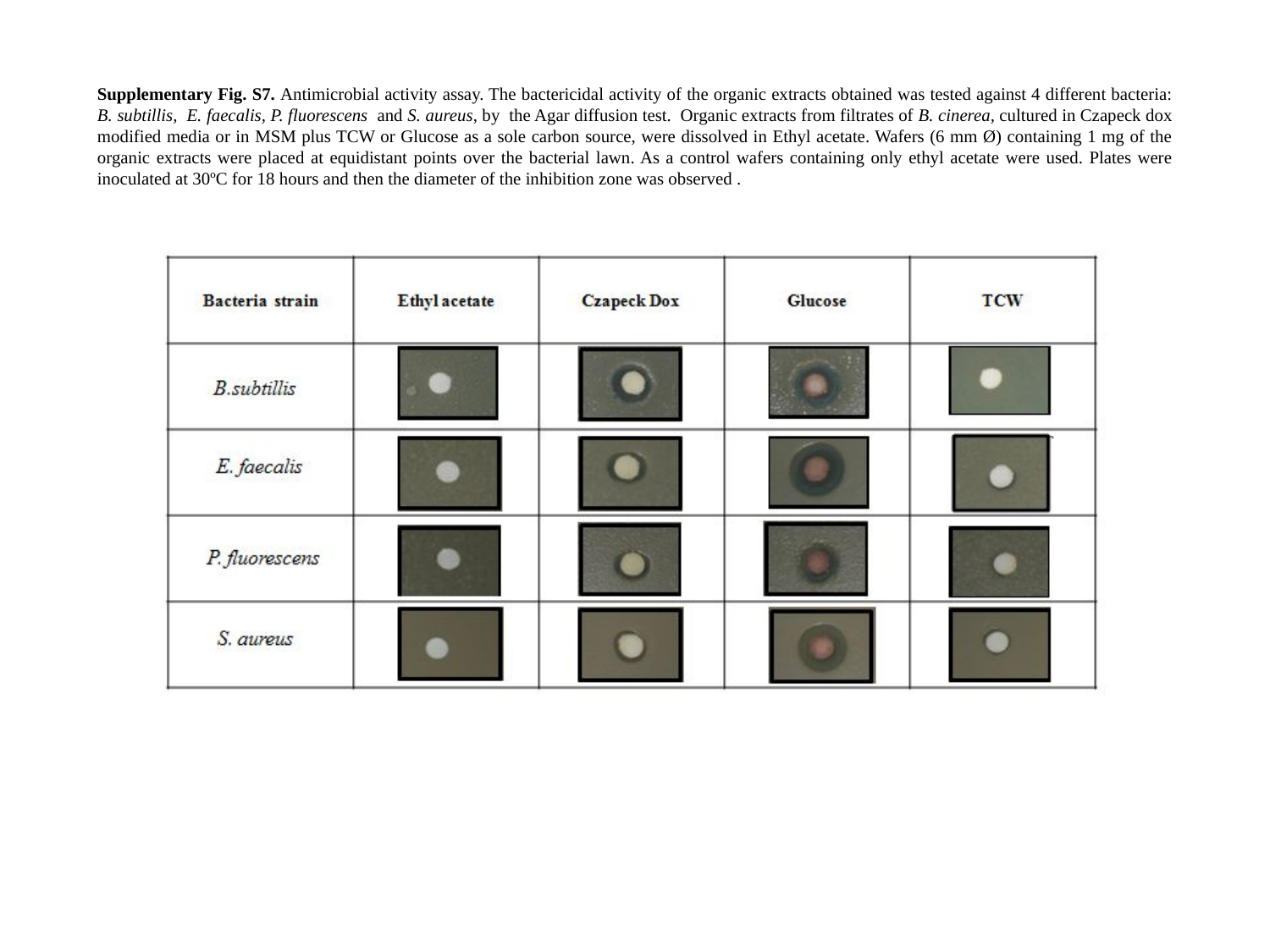

Supplementary Fig. S7. Antimicrobial activity assay. The bactericidal activity of the organic extracts obtained was tested against 4 different bacteria: B. subtillis, E. faecalis, P. fluorescens and S. aureus, by the Agar diffusion test. Organic extracts from filtrates of B. cinerea, cultured in Czapeck dox modified media or in MSM plus TCW or Glucose as a sole carbon source, were dissolved in Ethyl acetate. Wafers (6 mm Ø) containing 1 mg of the organic extracts were placed at equidistant points over the bacterial lawn. As a control wafers containing only ethyl acetate were used. Plates were inoculated at 30ºC for 18 hours and then the diameter of the inhibition zone was observed .

## Slide 10
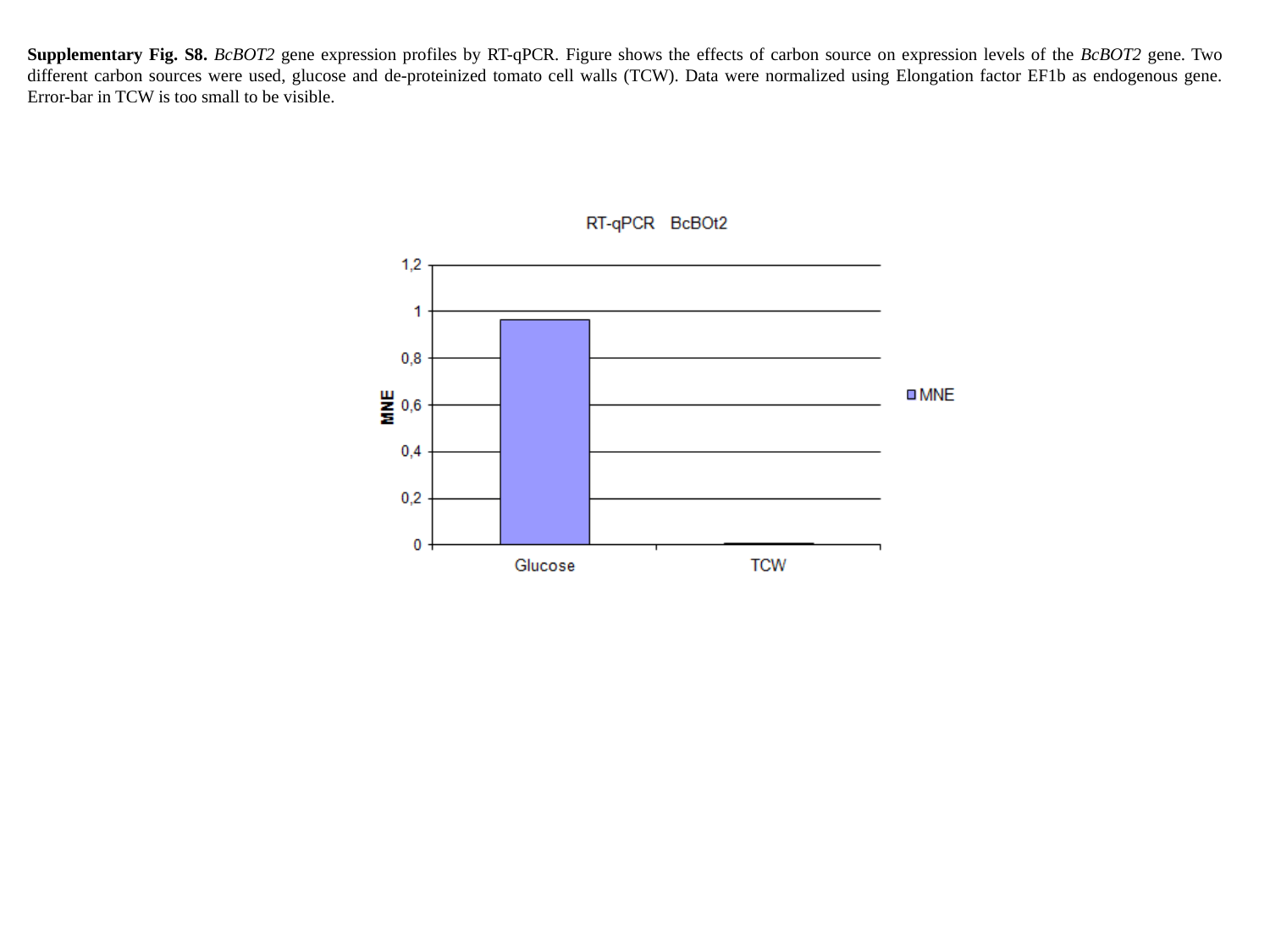

Supplementary Fig. S8. BcBOT2 gene expression profiles by RT-qPCR. Figure shows the effects of carbon source on expression levels of the BcBOT2 gene. Two different carbon sources were used, glucose and de-proteinized tomato cell walls (TCW). Data were normalized using Elongation factor EF1b as endogenous gene. Error-bar in TCW is too small to be visible.
